# Psychiatrization, assertions of epistemic justice, and the question of agency

**DOI:** 10.3389/fsoc.2023.1092298

**Published:** 2023-02-09

**Authors:** Jasna Russo

**Affiliations:** Department of Social Work, Alice Salomon University of Applied Sciences, Berlin, Germany

**Keywords:** madness, psychiatrization, first-person knowledge, epistemic oppression, testimonial injustice, hermeneutical injustice, co-optation

## Abstract

Thus far, the concept of epistemic injustice in the context of psychiatry has been discussed more widely by clinical academics than by authors with personal experience of psychiatrization. It is from the latter perspective that I critique the practice of attributing testimonial injustice solely to the “stigma against mental illness”, and point to psychiatric diagnosing itself as a principal enabler and re-producer of this form of injustice. In relation to hermeneutical justice, I take a closer look at initiatives seeking to incorporate (collective) first-person knowledge into the epistemic systems that currently dominate mental-health service provision and research. Highlighting the incompatibility of psychiatric knowledge claims with first-person ways of knowing, I discuss some of the issues and challenges involved in achieving epistemic justice for psychiatrized people and advancing our collective knowledge base. Finally, I turn to the questions of identity and agency in these processes.

## Introduction

My first encounter with the idea of epistemic injustice was at a conference about narrative approaches in healthcare.^1^ This concept, so simple and yet so profound, suddenly gave a name to the many struggles of psychiatrized people to have our knowledge count, not only in our individual lives, but also in our collective advocacy and research efforts. Engaging with the work of Fricker (2007) led me, a year later, to a conference called “Understanding Epistemic Injustice”.^2^ There, I realized how easy it is for a concept with the potential to become a change-making tool to be intellectualized to the point that it becomes an end in itself. Subsequently, as I began to investigate the use of “mad” people's testimonies in research, I pointed to the risk of overwriting and co-opting marginalized knowledge in the name of epistemic justice (Russo and Beresford, 2015; Russo, 2016). Unfortunately, this trend continues.

Thus far, the application of Fricker's work in psychiatric and mental health literature is characterized by descriptions of psychiatric patients' vulnerability to epistemic injustice, and by calls to “listen better” and “empathize more.” These approaches typically leave the concepts of “mental illness” or “psychiatric disorder” unquestioned (see e.g., Crichton et al., 2017; Kurs and Grinshpoon, 2017; Scrutton, 2017; Bueter, 2019; Grim et al., 2019; Drozdzowicz, 2021; Ritunnano, 2022). The biomedical framing of human crises and the practice of psychiatric diagnosing are hardly ever considered as a foundation of othering, or as principal enablers of epistemic (and other) injustice.

The notion of epistemic injustice has been less elaborated by psychiatrized people ourselves than by clinical academics. Yet, to those who have adopted it, it has proved helpful as a simple and convincing way to frame the disqualification of our knowledge and our truths that we face individually but also collectively, as organizations and movements (LeBlanc and Kinsella, 2016; Roper and Gooding, 2018; Russo, 2019; Todd, 2021; White, 2021; Daya, 2022).

Fricker's conceptualization of epistemic injustice is certainly worth refining, as it cannot be universally applied to all epistemic marginalization. Its most important strength lies in the ways in which different oppressed groups can develop and use this concept in their respective liberation struggles. The principal question, then, is how to work toward epistemic justice. Below, I discuss some issues pertaining to achieving epistemic justice for psychiatrized people, advancing our collective knowledge base, and strengthening our epistemic claims.

I structured the text following Fricker's differentiation of testimonial and hermeneutic injustice. At the end I briefly refer to the potential of Mad Studies as a project toward hermeneutical justice. Exploring that prospect in more depth would exceed the scope of this text, as its primary goal is to provide a critical perspective on the biomedicalized approaches to epistemic justice in the fields of psychiatry and mental health.

## Psychiatric diagnosing: The motor of testimonial injustice

Fricker states that “testimonial injustice occurs when prejudice causes a hearer to give a deflated level of credibility to a speaker's word” (Fricker, 2007, p. 1). In the psychiatric context, this form of injustice is often explained in terms of “the stigma of mental illness” to be resolved within individual encounters and through raising consciousness and empathy. A typical expression of this approach can be found in *The Routledge Handbook of Epistemic Injustice*, in the chapter that specifically addresses “mental illness.” It concludes that “[a]dopting an attitude of listening rather than ‘knowing best' would help to counter the stigma and sense of alienation and diminished agency that people with mental illness often experience” (Scrutton, 2017, p. 353). This suggestion can certainly do no harm. But can it, in the long term, counteract the amount of testimonial injustice that psychiatrically diagnosed people face? Scrutton's analysis, besides taking for granted the biomedical framing of “mental illness” as a health condition that some people simply have, also reduces testimonial injustice to (poor) clinical practice, to be remedied by improving relationships with patients. These presumptions detach the clinical encounter from its broader structural context and obviate the potential of the concept of epistemic injustice to bring about social change for psychiatrically diagnosed people.

Psychiatric diagnosing—whether subjectively experienced as helpful or oppressive—is not based on any replicable medical test (Kupfer, 2013), nor does it involve any consistent criteria or method. Yet it holds a massive amount of legal and social power. Kerstin Kempker, survivor of 3 years of forced detention and insulin shock treatment, reports:

“The diagnosis is the power tool of psychiatry. It suddenly changes everything. Diagnosis is the crime that deprives me of my freedoms – caringly, preventively and for my own good, of course. Without a diagnosis nobody would be allowed to do that to me. It would be deprivation of liberty, bodily injury and attempted murder. With a diagnosis of schizophrenia or endogenous depression, it is a medical treatment.” (Kempker, 1997, p. 69, own translation).

Not all diagnoses can elicit forced treatment, but treatment cannot be forced without a psychiatric diagnosis. It has been established that the diagnoses with the most power to coerce disproportionally land on multiply oppressed people and decisively depend on the social location from which they come into contact with services. The diagnosis of psychosis, for example, is given three to four times more often to African Americans than to Euro-Americans (Schwartz and Blankenship, 2014). Black people in England are almost five times as likely as white people to be detained under the Mental Health Act, and community treatment orders are imposed on “Black or Black British” people more than ten times as often as on white people (NHS Digital, 2021).

Psychiatric diagnosing readies entire social groups—and some far more than others—to routinely become subject to many subsequent wrongs. People labeled mentally disordered or ill are therefore not only *vulnerable* to testimonial injustice, but are being *systematically made into its objects*. And this practice, far from being obsolete, is currently taking place all over the globe. This well-organized and deep-rooted cycle of injustice is unlikely to be halted by improved and humanized encounters with individual clinicians. This view of epistemic injustice might correspond to Fricker's assertion that, in distinction to hermeneutical injustice, “the wrong of testimonial injustice is always inflicted from individual to individual” (Fricker, 2007, p. 138). Even though this is ultimately the case within all social interactions, it does not mean that testimonial injustice resulting from the ongoing psychiatrization of particular lives should be treated as an interpersonal matter only.

In their analysis of how legislation and the mental health paradigm work in synergy, Beaupert (2018) states:

“[T]he medico-legal discourse of mental health laws, by consecrating this symbolic violence, operates to manipulate and nullify individual ways of knowing and being, and to radically diminish opportunities for the epistemologies of users and survivors to exert influence on societal systems and structures. Constructions of people with psychosocial disability as lacking capacity and ‘insight' are central to these processes of dehumanization.” (p. 16)

Beaupert's analysis makes clear that vulnerability to injustice arises, not from “mental illness”, but from organized societal responses to what is labeled as “mental illness”. It also demonstrates the need to change laws and abolish practices that enable and sustain testimonial injustice. Such a project goes far beyond improving clinicians' attitudes or collecting more evidence that testimonial injustice occurs within psychiatry. It requires political will and committed work on different levels and from many social actors. The *Convention on the Rights of Persons with Disabilities* (United Nations, 2007), as the first international treaty to prohibit forced detention and treatment based on psychiatric diagnosis, offers a good framework to underpin and lead such action (Minkowitz, 2007, 2010).

## Knowledge claims of people deemed mad and struggles for ownership

According to Fricker, hermeneutical injustice occurs “when a gap in collective interpretive resources puts someone at an unfair disadvantage when it comes to making sense of their social experiences” (Fricker, 2007, p. 1) Following up this framing, I wish to discuss two closely intertwined issues regarding the (collective) knowledge of people deemed mad: the presumption of our inability to (collectively) articulate what we experience and what we know; and the question of who qualifies to work toward hermeneutical justice. Together, these two issues form a tight knot that is paradigmatic to the disciplines of psychiatry and mental health: speaking on behalf of others on the presumption that they are unfit to do so, and then taking over their agenda and acting in their name. This deeply rooted attitude normalizes a wide variety of practices, ranging from overt control and patronization to subtle forms of silencing that are much harder to challenge, as they appear supportive (Russo, 2012; Dimitrova, 2021).

The division between a hard-to-comprehend *them* who need skilled and knowledgeable *us* to put forward their epistemic claims is enshrined in the work of various experts (see for example Estroff, 1981, 2004; Hornstein, 2009). The fundamental contradiction between the declared aims of such undertakings and their ethics and methodologies is rarely at issue, including for those whose marginalized ways of knowing are at stake. Some authors argue that mental health professionals might need to provide “patients” with resources and tools to help them express their experiences, even while recognizing the risk of secondary epistemic injustices in such attempts; first-person reports can be “misdescribed or forced into imposed categories” (Drozdzowicz, 2021, p. 4). The suggested solution here is to develop phenomenological tools jointly with “patients” as well as tailor them to specific “mental illnesses” (Drozdzowicz, 2021). Such “biomedicalized participatory practices” (de Boer, 2021), and their repeated failure to uphold the distinctiveness of marginalized perspectives within the established hierarchies of knowledge, have already been documented and analyzed in the context of psychiatric and mental health research and praxis (Davidow, 2013; Staddon, 2013; Brown and Stastny, 2016; Carr, 2016, 2018, 2019; Fabris, 2016; Penney and Prescott, 2016). This body of critical work, mainly created by authors with first-hand experience of psychiatrization, offers important insights into how efforts to integrate first-person knowledge, in order to transform dominant structures of both mental health service provision and knowledge production, often end up sustaining those structures and ultimately reproducing inequalities.

Kristie Dotson's concept of “irreducible epistemic oppression” (Dotson, 2014) offers a helpful framework to further understand the incompatibility of psychiatric knowledge claims with collective first-person ways of knowing. Dotson identifies a specific form of epistemic oppression “that is not solely reducible to social and political factors but rather follows from a feature of epistemological systems themselves, that is epistemological resilience” (Dotson, 2014, p. 116). In their view, this form of oppression “can only begin to be addressed through recognition of the limits of one's overall epistemological frameworks” (2014, p. 116). Acknowledging such limits is rarely a viable option in the official knowledge production of a field that is on all levels (including funding) dominated by the biomedical model of mental illness. Efforts toward hermeneutical justice in psychiatry are therefore limited to attempting to upgrade the biomedical framework by incorporating “lived experience” as a historically missing perspective. While the absence of first-person knowledge is increasingly being identified, the distinctiveness of this epistemic source is not recognized—and its crucial mismatch with the dominant methods of knowledge-making on madness and distress is not being adequately addressed (Rose et al., 2018).

From the onset of psychiatry, those considered to be of “unsound mind” have not only generated and articulated our knowledge but have also documented it in various formats. Besides different oral traditions, the written sources include numerous biographical accounts and collections of essays, petitions, position papers, research reports, concepts of support and theoretical contributions.^3^ However, this considerable body of knowledge is rarely explored on its own merits or given a chance to deepen and advance its own epistemology. When considered at all, our accumulated knowledge is likely to be seen only in connection with psychiatry and adapted to that context as a matter of course—even though it largely emerges in resistance to, and as an act of liberation from, that very context. This re-psychiatrization of first-person labor (both individual and collective) takes over the ownership of our knowledge and suppresses our agency as knowers. Regardless of its intentions, the continuous process of co-optation distorts and de-politicizes crucial aspects of this epistemic source that reach beyond the topics of madness and psychiatry and encompass relevant and valuable understandings of the world we live in. These circumstances turn Fricker's question about the collective capacity to articulate certain experiences into the question of *who* is entitled and resourced to work with those articulations, and in what kind of process.

The initiatives to include our knowledge—from consultancy to collaboration and coproduction—have thus far been restricted to the fields of psychiatric and mental health research. The hegemonial discourse of these fields channels all knowledge production, including inquiries of alternatives to psychiatry, into an ongoing dialogue with the biomedical model. The implicit demands of such environment impose firm limits on *what* can be researched and dictate *how* evidence-making should ensue (Faulkner, 2015; Russo, 2018). The collective first-person knowledge of people deemed mad transcends both the research questions and the methodologies of psychiatric and mental health research. The narrow focus of these research areas means that any attempt to subsume our knowledge, will inevitably miss crucial parts of that knowledge. To explore and deepen this comprehensive body of work with the respect which is its due requires a different epistemological framework. It is unlikely for such a framework to emerge within disciplines that were founded on the denial of “mad” people's rationality and remain reluctant to make room for our perspectives. There is dispute about the accomplishments of the past few decades of attempts (in Western countries) to bring our collective knowledge into a “science” that is used to study and treat us as its objects (Staddon, 2013). In these countries, the intellectual labor of “lived experience experts” is likely to be funded and supported only to enrich the dominant model of “mental illness”, create better quality knowledge about “us”, and improve treatments we supposedly need. This type of inclusion can foster the individual academic careers of people deemed mad but, in the long term, it actively delays and hinders our own theory-building and prevents us from creating sustainable structures to connect our work internationally and globally.

Fricker (2007) writes that “hermeneutical injustice, whether incidental or systematic involves no culprit” and that “no agent *perpetuates* hermeneutical injustice – it is a purely structural notion” (p. 158, emphasis in original). Leaving aside a debate about whether any human interaction can be of a solely structural or individual nature, what are the practical implications of this kind of framing in the context of official knowledge production on madness and distress? If no culprit is involved, how can we ever address hermeneutical injustice, particularly in projects that seek to involve first-person knowledge-holders within (Eurocentric) psychiatric and mental-health disciplinary frameworks, and on their terms?

## Closing remarks

Even though the above exploration of the ways in which the concept of epistemic injustice is being considered in the fields of psychiatry and mental health is neither systematic nor complete, some general trends can be noticed. Testimonial injustice is mainly seen as intrinsic to “mental illness” and is commonly approached in terms of quality of contact with “patients.” There is little willingness to question the role of the psy-complex^4^ *per se* in the making of “psychiatric patients”, and stop the practices of its professions that are foundational to testimonial injustice. At the same time, there is a growing eagerness to include “lived experience expertise” in mental health and even take on the task of articulating collective first-person knowledge. Such initiatives are not necessarily framed as work toward hermeneutical justice, but often do claim to foster marginalized knowledge. In the above section I have tried to highlight some of the fundamental contradictions intrinsic to these undertakings.

Finally, I'd like to open the question about the implications of identity in hermeneutical justice work. Psychiatrization intersects with the rest of our (unequal) lives and affects us differently. Also, whether being imposed, accepted or reclaimed in the psychiatric context, our diverse identities are fluid, and more often something to leave behind rather than hang on to or ontologize. But can the question of whether or not one has experienced psychiatrization be rendered irrelevant, or even secondary, in the attainment of hermeneutical justice? Has the work of finding common ground, understanding and politicizing oppression, and claiming rights ever been carried out by anybody other than those who have been subjected to that form of oppression? And why does something so obvious prove hard to respect in the case of people deemed mad or declared mentally ill? There are many social justice issues, both within and outside of the realm of psychiatry, that we should all stand up for. But when it comes to particular ways of knowing, standing up for justice might mean deliberately standing aside from, rather than in the way of, knowledge that has been silenced for so long and which seeks to find and articulate itself.

As stated above, in comparison to the number of publications by mental health and other experts, there is only a small number of references to epistemic injustice by authors whose own psychiatric experience is integral to their work. But already this body of work displays a different uptake of Fricker's concept—one which transgresses clinical context and positions psychiatrization within the broader human-rights framework (LeBlanc and Kinsella, 2016; Roper and Gooding, 2018; Todd, 2021; White, 2021; Daya, 2022). In this text I narrowly focused on the particular concept of epistemic injustice, but there are many more authors who address this same phenomenon using different terminology—such as for example, “psychiatric disqualification” (Carr et al., 2017, 2019).

To me, advancing our collective first-person ways of knowing is a matter of ethics (Russo, 2021), methodologies and, not least, independence from the psy-complex. The future will show whether Mad Studies, as a form of activist scholarship that seeks to flip the microscope away from “madness” (Costa, 2014) and to dismantle whiteness as norm (Gorman, 2013; Gorman et al., 2013; Eromosele, 2021; Joseph, 2021; King, 2021; Sharma, 2021), is up to such a task. In the meantime, I wish for Mad Studies to keep fostering hermeneutical justice—not as a desirable nor once-and-forever achievable state, but as an ongoing process which never shies away from taking an honest look at itself; which resists the seductiveness of having the last word; and which always stays open to those who have not yet spoken.

## Data availability statement

The original contributions presented in the study are included in the article/supplementary material, further inquiries can be directed to the corresponding author.

## Author contributions

The author confirms being the sole contributor of this work and has approved it for publication.

## Footnotes

^1^A Narrative Future for Health Care. London (2013). Available online at: https://medicalhumanities.wordpress.com/2013/05/07/a-narrative-future-for-healthcare-international-conference-guys-hospital-campus-of-kings-college-london-june-19-21-2013/.

^2^ http://www.bristol.ac.uk/philosophy/research/epistemic-injustice-/

^3^A selection of sources between 1620 and 2008 was assembled by The Opal Project: “Ourstory of Commitment: A living history.” Available online at: http://www.theopalproject.org/ourstory.html.

^4^For the explanation of psy-complex see https://www.encyclopedia.com/social-sciences/dictionaries-thesauruses-pictures-and-press-releases/psy-comple.
